# Supplementary material for: The Characteristics and Expression Analysis of the Tomato SlRBOH Gene Family under Exogenous Phytohormone Treatments and Abiotic Stresses
Source: Int J Mol Sci. 2024 May 26;25(11):5780. doi: 10.3390/ijms25115780 (PMC11171631; doi:10.3390/ijms25115780)
Supplement: Supplementary file 1 [file ijms-25-05780-s001.zip › Figure S1.pdf]

The corresponding IC_{50} curves were as follows:

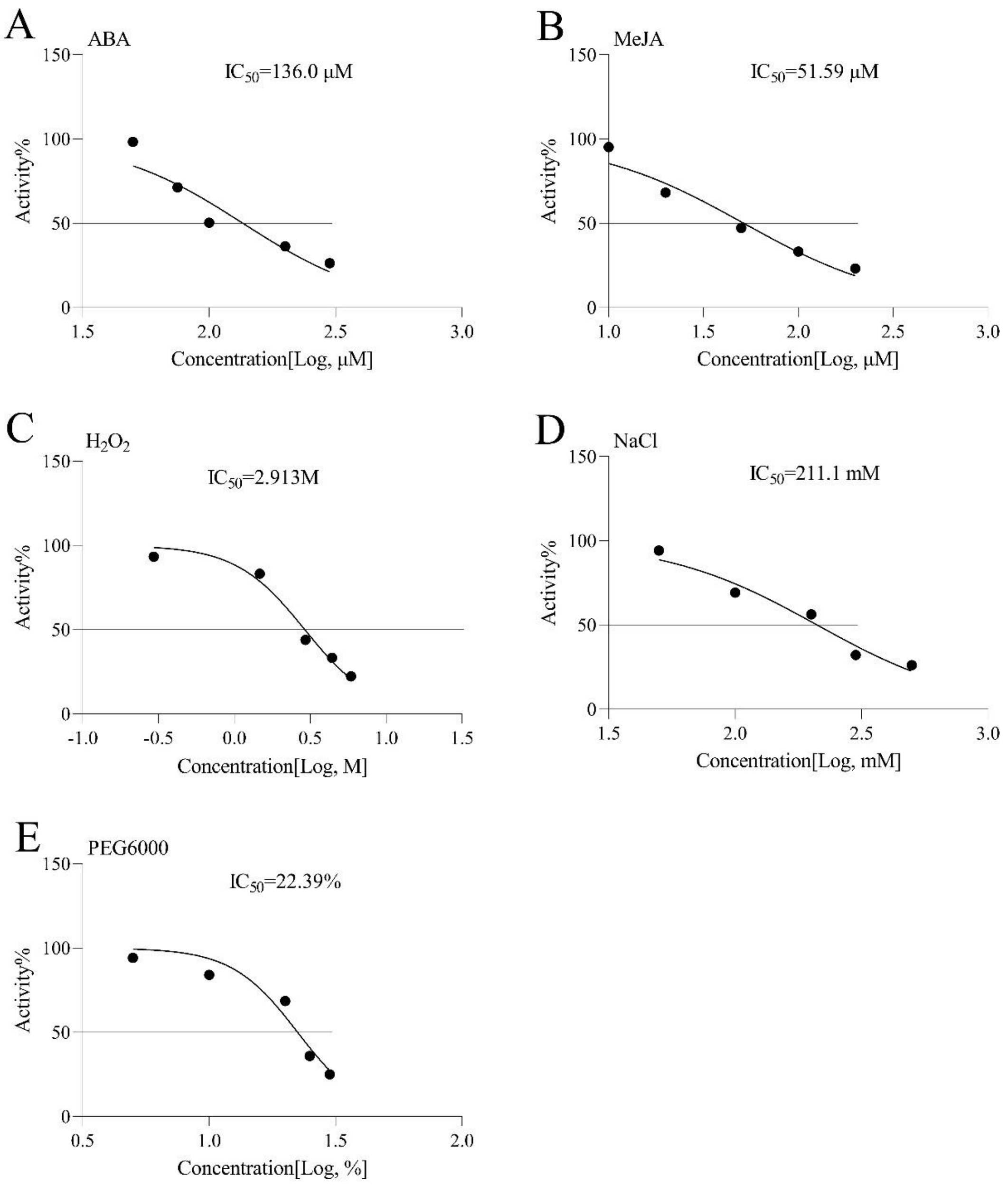

Figure dose-response curves, Y-axis is Relative cell viability of tomato seedlings (Activity, %). X-axis is the logarithmic value of A (ABA), B (MeJA), C (H2O2), D(NaCl)，and E (PEG6000) concentration.
