# Supplementary material for: The Characteristics and Expression Analysis of the Tomato SlRBOH Gene Family under Exogenous Phytohormone Treatments and Abiotic Stresses
Source: Int J Mol Sci. 2024 May 26;25(11):5780. doi: 10.3390/ijms25115780 (PMC11171631; doi:10.3390/ijms25115780)
Supplement: Supplementary file 1 [file ijms-25-05780-s001.zip › Table S1.pdf]

Table S1. Primers for qRT-PCR for expression analysis of the *SlRBOH* family genes in tomato.

Gene name	Primer sequence (5’ to 3’)	
SlRBOH1	F: CCTAACCATACGCTTGGCTCACAG	R: GCACGAGCAGCACCAGACTTAC
SlRBOH2	F: GGACCGCTGAACAAACGAGGAG	R: AGCCACAGAGTCTTCACGAACATC
SlRBOH3	F: TCTACTGTGGGATGCCTGTCTTGG	R: TGTGGAACTCGAAACGTGTGGATG
SlRBOH4	F: TTTGACTGGTGTTGAGGGCATCAC	R: CTCCTCCTGAAGCGTCGTGTTG
SlRBOH5	F: AGAAGCCAACCGCATCCTCCTC	R: ATCCAGAGCCACCATCAGTCCTAC
SlRBOH6	F: CAAGAATGGGGCGGATGGAATCAG	R: ATCTCCCTCCTTCCCTGTGGTTG
SlRBOH7	F: AAGAGGAAGCACCGCCCAATG	R: CGATGCTGCTGCCGATGTC
SlRBOH8	F: GAAGAGGAAGCACCGCCCAATG	R: CCGATGCTGCTGCCGATGTC
SlRBOH9	F: GAGTCGGTATTGGTGGAGCATCG	R: TCGCTTTCGCAACAGCCTCAG
SlRBOH10	F: GGTCGGTATTGGTGGAGCATCGC	R: CGCTTTCGCAACAGCCTCAGC
SlRBOH11	F: TCCTTCACACTGGCAACACATTCG	R: AGCAGCCGTGGAGGATCAAGAG
SlActin	F: AATGAACTTCGTGTGGCTCCAGAG	R: ATGGCAGGGGTGTTGAAGGTTTC
